# Development of the OnTrack Diabetes Program

**DOI:** 10.2196/resprot.2823

**Published:** 2015-05-26

**Authors:** Mandy Cassimatis, David J Kavanagh, Andrew P Hills, Anthony C Smith, Paul A Scuffham, Steven Edge, Jeremy Gibson, Christian Gericke

**Affiliations:** ^1^Queensland University of TechnologyInstitute of Health and Biomedical InnovationBrisbaneAustralia; ^2^The Wesley Health and Medical Research InstituteLevel 8 East Wing, The Wesley Hospital451 Coronation Drive, Auchenflower, 4066BrisbaneAustralia; ^3^Queensland University of TechnologyInstitute of Health and Biomedical InnovationLevel 4, 60 Musk Avenue, Kelvin Grove, 4059BrisbaneAustralia; ^4^Mater Mothers' Hospital, Mater Medical Research InstituteLevel 3 Aubigny PlaceRaymond Terrace, South Brisbane, 4101BrisbaneAustralia; ^5^Griffith Health InstituteGriffith UniversityBrisbaneAustralia; ^6^University of Queensland Centre for Online HealthGround floor, Main Building, Princess Alexandra HospitalWoolloongabba, 4102BrisbaneAustralia; ^7^Griffith UniversitySchool of Medicine & Griffith Health InstituteUniversity Drive, Meadowbrook, 4131BrisbaneAustralia

**Keywords:** type 2 diabetes, depression, anxiety, self-management, Internet, online, intervention, randomized, protocol

## Abstract

**Background:**

Type 2 diabetes affects an estimated 347 million people worldwide and often leads to serious complications including blindness, kidney disease, and limb amputation. Comorbid dysphoria is common and is an independent risk factor for poor glycaemic control. Professional support for diabetes self-management and dysphoria has limited availability and involves high costs, especially after regular hours, and in rural and remote areas. Web-based cognitive behavior therapy offers highly accessible, acceptable, and cost-effective support for people with diabetes. This paper describes the development of OnTrack Diabetes, a self-guided, Web-based program to promote improved physical and emotional self-management in people with Type 2 diabetes.

**Objective:**

The objective of the study is to describe the development of the OnTrack Diabetes program, which is a self-guided, Web-based program aimed to promote euthymia and improved disease self-management in people with Type 2 diabetes.

**Methods:**

Semistructured interviews with 12 general practitioners and 13 patients with Type 2 diabetes identified enablers of and barriers to effective diabetes self-management, requirements for additional support, and potential program elements. Existing resources and research data informed the development of content, and consultants from relevant disciplines provided feedback on draft segments and reviewed the program before release. Using a self-guided delivery format contained costs, in addition to adapting program features and modules from an existing OnTrack program.

**Results:**

A separate paper describes the protocol for a randomized controlled trial to provide this required evaluation.

**Conclusions:**

Development of the OnTrack Diabetes program demonstrates strategies that help ensure that a program is acceptable to users. The next stages involve testing users’ experiences and examining the program’s effectiveness and cost-effectiveness in randomized controlled trials.

**Trial Registration:**

The Australian New Zealand Clinical Trials Registry (ACTRN): 12614001126606; https://www.anzctr.org.au/Trial/Registration/TrialReview.aspx?ACTRN=12614001126606 (Archived by WebCite at http://www.webcitation.org/6U0Fh3vOj).

## Introduction

### Type 2 Diabetes Self-Management

Type 2 diabetes is a burgeoning epidemic that affects an estimated 347 million people worldwide [1], and is becoming one of the leading causes of global disease burden [1]. Inadequate diabetes self-care is strongly associated with poor glycaemic control [2,3], which increases the risk of diabetes complications including peripheral limb amputation, blindness, and end-stage renal disease [1], as well as cardiovascular disease and stroke [4]. A 21% decrease in the incidence of diabetes complications occurs with each 1% improvement (reduction) in glycosylated haemoglobin A1c level [5], which indicates the utility of improving diabetes self-management. However, patients often struggle to meet recommended treatment targets and find it difficult to implement the behavioral changes required to achieve such improvements.

Diabetes patients are two to three times more likely than people without diabetes to experience depression, anxiety, stress, and reduced well-being [6-8]. Dysphoria appears to be both a consequence of Type 2 diabetes and to have a role in the condition’s pathogenesis [9], impairing glycaemic control both directly via physiological mechanisms, and indirectly via reduced diabetes self-care [10,11]. As a result, dysphoric patients have an increased risk of diabetes complications [12,13] and premature mortality [14]. Optimal diabetes management therefore requires that both mood and behavioral disease self-management be targeted.

Controlled trials of diabetes self-management interventions have shown that effective components include diabetes education [15,16], promotion of adherence to blood glucose self-monitoring [17,18], physical activity [19,20], dietary [21], and medication regimes [22], and emotional support [23]. Interventions that incorporate only behavioral components have generally failed to produce robust and sustained improvements in psychological and emotional outcomes [23]. Similarly, interventions that specifically target depression or anxiety have typically failed to produce substantial improvements in diabetes self-management and physical outcomes [24]. Even for high-functioning individuals, the complexity of the Type 2 diabetes treatment regime exposes patients to a range of daily physical and emotional challenges [8]. A holistic intervention that incorporates both behavioral and psychological support may therefore offer optimum efficacy.

While some key components of effective support for Type 2 diabetes self-management have been identified, health system limitations prevent their reliable provision [25], especially after regular hours or in more remote locations, where greater population spread and reduced practitioner to population ratios conspire to reduce access. Diabetes self-management support services that offer wide outreach and cost-effectiveness are needed.

### Web-Based Programs for Type 2 Diabetes

Over recent years, Web-based interventions, and in particular those based on cognitive-behavior therapy (CBT), have produced substantial improvements in emotional and behavioral outcomes in a range of problem areas [26], with effects similar in size to those of face-to-face treatments [27]. CBT-based Type 2 diabetes interventions similarly have produced significant improvements in diabetes self-care [28,29], and psychosocial outcomes. These programs have also shown high user uptake, acceptability, and usability, even in older users [30].

Globally, Web access is increasing rapidly; with the proliferation of cable and mobile networks increasingly bridging geographical and even socioeconomic divides [31]. Web-based delivery of intervention programs may assist with meeting the need for improved access to additional disease self-management support for people with Type 2 diabetes [25], conveying the advantages of 24-hour availability, broad access, privacy, and lack of stigma. Self-guided programs also show steeply falling unit costs as user numbers increase.

Web programs based on empirically well-established theories have shown superior efficacy in improving diabetes self-management outcomes compared with programs that do not have a strong theoretical and empirical basis [28]; in particular, chronic disease self-management programs that use social cognitive theory (SCT) [31] as their theoretical underpinning have demonstrated efficacy [32]. SCT is appropriate to chronic disease self-management intervention, as it specifies predictors of human motivation and behavior that can be targeted in self-management [33], including specific skills, self-efficacy [32,34], goals, and self-administered incentives [31]. SCT encourages patient empowerment, positing that humans actively make sense of the world and shape their own experiences, giving them the capacity to exercise choice and change their behavior. The theory holds that environmental, interpersonal, and intrapersonal variables are interlocked in processes of reciprocal determinism. Research that demonstrates that diabetes self-management [35-38] and mood [39,40] have strong associations with cognitive and psychosocial factors is consistent with this view, and lends support to diabetes interventions being based on SCT principles.

While Web-based CBT has shown efficacy in reducing depression and anxiety symptoms in people with diabetes [41], interventions primarily focused on targeting mood have yielded mixed results in terms of their effects on glycaemic control [42]. Similarly, behaviorally focused Type 2 diabetes interventions have demonstrated improved glycaemic control and behavioral outcomes, but they do not typically produce substantial differential improvements in psychological well-being [28]. Programs that simultaneously address behavioral aspects of Type 2 diabetes self-care are needed [42]. Such interventions would be appropriate for implementation in the mainstream Type 2 diabetic population and may support those experiencing primarily psychosocial barriers to self-care, as well as those with co-occurring distress.

Most current Web-based CBT interventions are guided programs that incorporate support from a health professional [43,44]. However, studies that compare guided CBT-based programs with minimal support have similar impacts on clinical [44,45] and behavioral [46] outcomes, as well as user engagement [47]. Self-guided Web-based interventions have shown equal effectiveness to guided interventions [48] and offer the advantages of self-paced learning and skill acquisition, and higher perceived autonomy and privacy. Further, Web-based programs encourage users to adopt an independent role in their disease management, which may enhance patient empowerment. There remains a need for further research on self-guided, Web-based Type 2 diabetes self-management programs that incorporate mood support.

This paper describes the development of the OnTrack Diabetes program [49], which attempts to address the need for a Web-based program that targets both Type 2 diabetes self-management and dysphoria. SCT [31] and Elaborated Intrusion Theory [50-52] inform OnTrack Diabetes, which incorporates both CBT and motivational strategies. The program is designed to provide a holistic approach to improving Type 2 diabetes self-management and mood, and to endorse user empowerment by encouraging users with diabetes to take an autonomous role in managing their condition; it can also be used in either a self-guided or therapist-assisted mode. Practitioners can also use the program to guide sessions supporting patients’ self-management; they are given a separate log-in.

## Methods

### Development of the OnTrack Diabetes Program

#### Step 1. Qualitative Research

**Figure 1:**
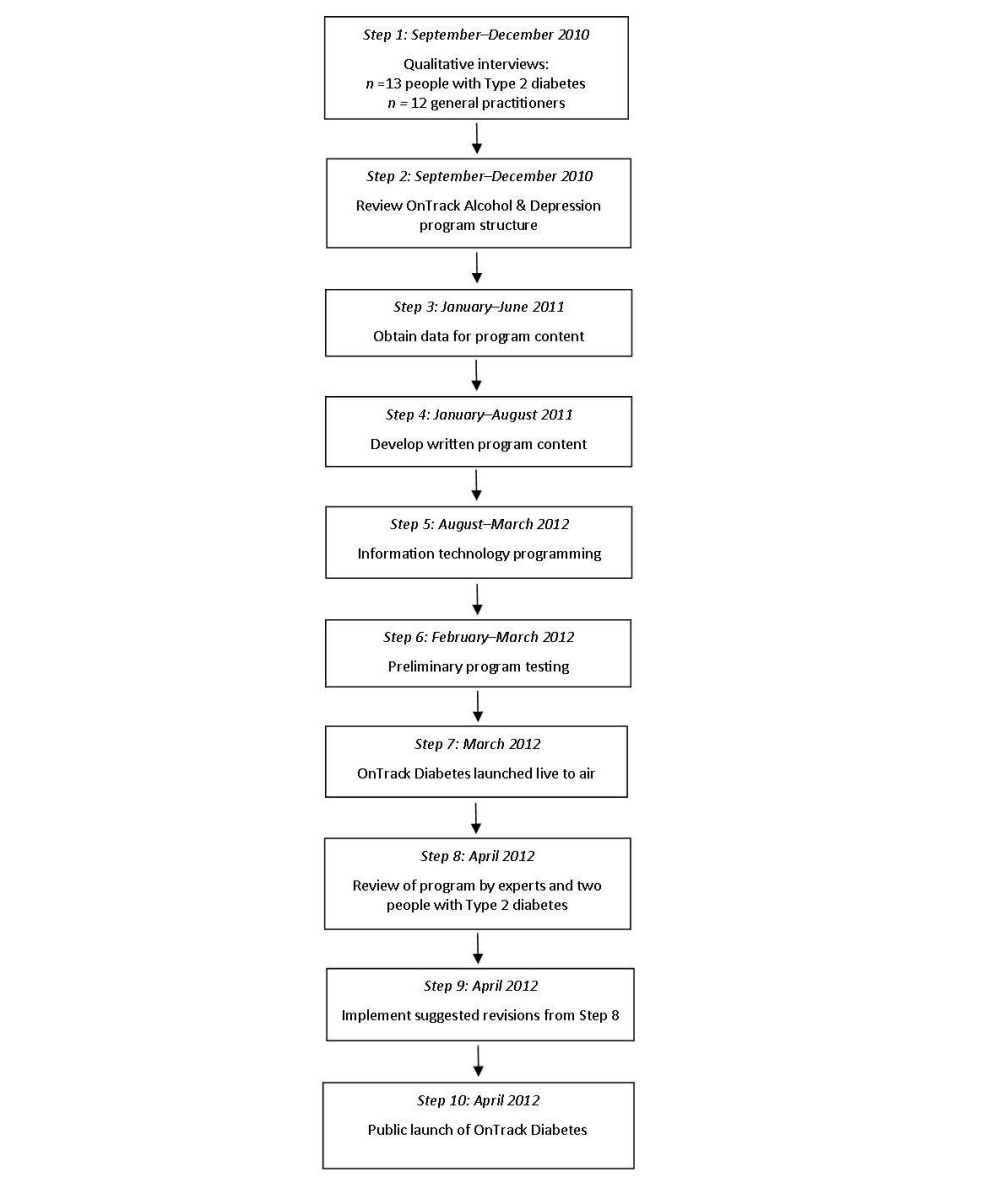
Steps involved in OnTrack Diabetes program development.

Figure 1 shows the Steps in the development of the program. Semistructured interviews were conducted to explore enablers and barriers associated with effective Type 2 diabetes self-care, together with diabetes-related emotional challenges, requirements for additional disease management support, and suggestions for elements in an online support program. The sample comprised 13 people with Type 2 diabetes and 12 general practitioners (GPs). GPs were asked the circumstances in which they would refer patients to an online Type 2 diabetes self-management support program, and the factors that may inhibit patient referral. Results revealed that patients and GPs shared most perspectives on diabetes self-management. Both the patients with diabetes and GPs identified a need for additional informational, motivational, emotional, and social support. Suggestions for program content included self-monitoring tools, informational support, motivational assistance with improving and maintaining physical activity and diet, goal setting assistance, progress feedback, social support via a chat room and accessibility to health professionals. Detailed results are available in a separate paper [53].

#### Step 2. Basic Structure and Functionality

The existing OnTrack Alcohol and Depression program [54] was proposed as a basis for the layout of OnTrack Diabetes, and the appropriateness of this was confirmed by a review of its structure. Motivational videos, mindfulness and relaxation audios, and information technology coding from some of the self-monitoring and program tools were adapted for use in OnTrack Diabetes.

#### Step 3. Assembly of Information Resources

Sources that informed the development of OnTrack Diabetes information resources and tools included the Diabetes Australia guidelines for Type 2 diabetes management [55]; National Health and Medical Research Council Physical Activity and Nutrition Guidelines for Australian Adults [56]; Optometrists Association Australia [57]; Australasian Podiatry Council [58]; Medicare Australia [59], and relevant peer-reviewed empirical literature. A nutritionist, ophthalmologist, and podiatrist were consulted to discuss proposed content.

#### Step 4. Content Development

MC compiled the obtained information and discussed proposed content inclusions with DK. The program content addressed the barriers to Type 2 diabetes self-care identified in qualitative research, and attempted to maximize enhancers. Information resources complement the program’s interactive tools and provide the impetus for goal setting and planning, while providing material that can be integrated into primary care. For example, the “My Feet Check” resource contains a diagram of feet on which the date and any changes can be marked, and a checklist to tick off symptoms that can be taken to podiatry appointments.

#### Step 5. Programming

OnTrack Diabetes information technology programming logic is based on eXtensive Markup coding developed for OnTrack Alcohol and Depression by SE and JG. In collaboration with them, MC coded tools and guidebook pages for the site. Programming modifications and the development of new features exclusive to OnTrack Diabetes was then undertaken. The administration site was built to include functions specific to this trial, including data recording and storage, access to study measures, and a schedule of follow-up study measure reminders. A graphic designer designed the website interface, inserted relevant images, and formatted the program.

#### Step 6. Preliminary Testing

Both the information technology programmers and external observers tested OnTrack Diabetes several times for bugs, errors in functionality, and design issues.

#### Step 7. Test of the Live Program

OnTrack Diabetes then had a soft initial launch to enable further screening for bugs and tests for functionality by MC and programmers.

#### Step 8. Expert Review

An endocrinologist and diabetes educator were invited to provide feedback on OnTrack Diabetes’ contents, and AH, AS, PS, and two people with Type 2 diabetes who participated in the qualitative interviews (Step 1) also reviewed the program and provided feedback.

#### Step 9. Program Revision

The program content was revised in response to the reviews that were undertaken in Stage 8. Specifically, some information fact sheets were added, including on providing ideas for safe physical activity for individuals with limited physical capacity. Further, modifications were made to the program’s information technology functionality, as the reviewers had provided feedback regarding bugs that they had found while testing the site’s tools and resources.

#### Step 10. Launch and Efficacy Trial

A randomized controlled trial was commenced with potential participants registering interest on the site’s home page.

### OnTrack Diabetes Program Content

#### Key Elements

**Figure 2:**
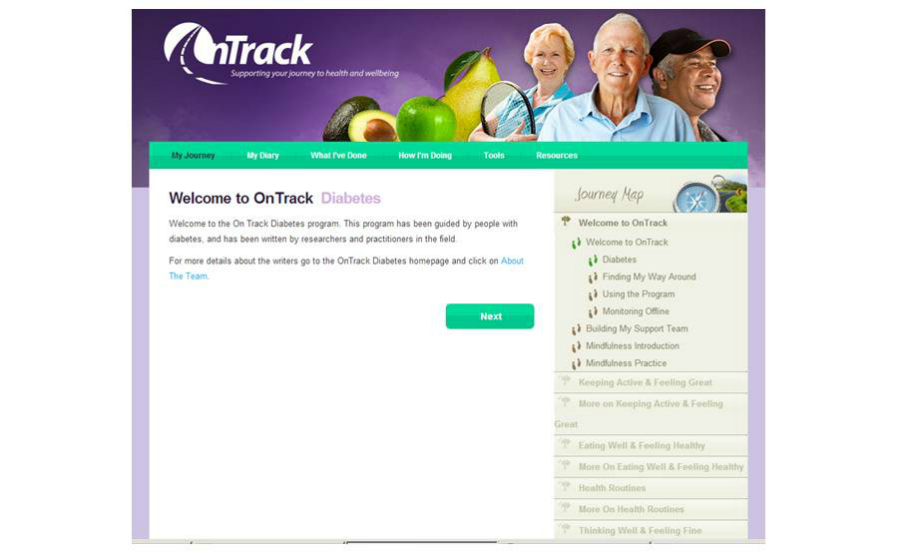
Screenshot of the OnTrack Diabetes program layout.

Figure 2 shows the initial screen in the program, which includes the key elements.

#### Self-Monitoring and Goal Attainment Scaling

**Figure 3:**
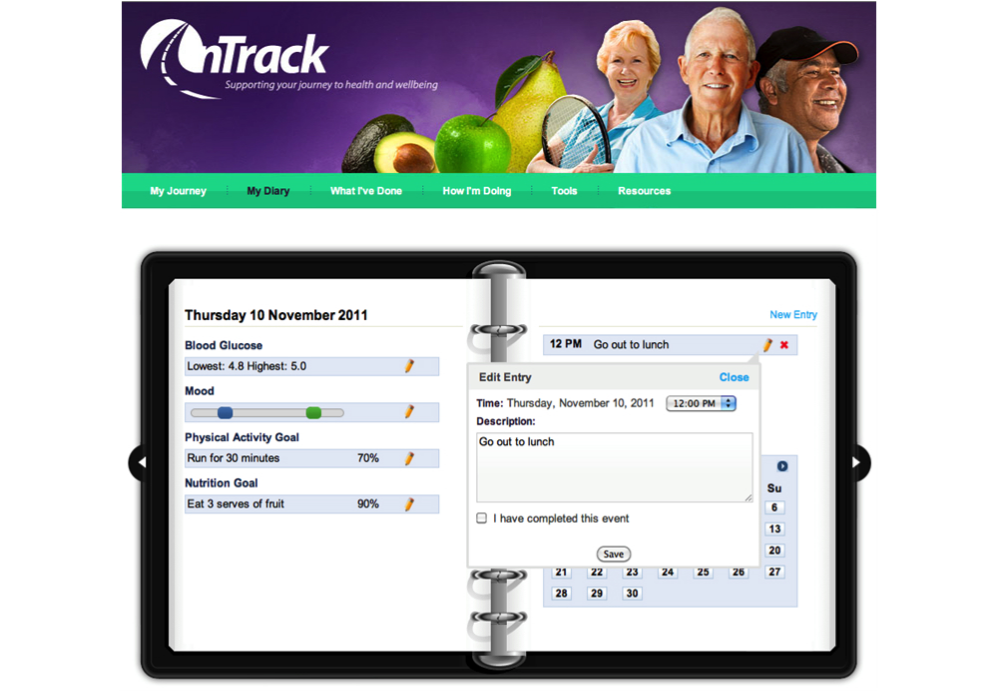
Diary for self-monitoring.

The “My Diary” tab provides an electronic self-monitoring record of daily goal attainments in relation to physical activity, eating, and health routines (on a sliding scale from 0% to 100%); highest and lowest blood glucose levels; and mood (on a scale from best to worst). Figure 3 shows a diary page. Entries are represented in feedback graphs that are shown under the “How I’m Doing” tab. The graphs display averages per day over the previous month, and averages per week over the previous 3 months, for each self-monitoring area. Users are encouraged to recognize correlations between the different outcomes, in order to better understand their interrelationship and how they can improve their diabetic and dysphoria control. The monitoring and feedback functions emphasize the SCT focus on goals and on the motivational effects of feedback on goal attainment.

#### Resources

At the top right of each screen, a “Resources” tab provides access to over 40 fact sheets and quizzes on diabetes and its management, and also to 8 mindfulness audios to guide practice sessions, which can be accessed on the computer as audios or text, or downloaded onto mobile phones in the MP3 format.

#### Journey Map

Based on our previous research on user preferences concerning Web program formats [60], modules in OnTrack Diabetes are available in any order and at any time, using the tabs shown under the “Journey Map” at the right hand side of Figure 2. All of the tools within the program are also available at any time under the respective tabs at the top of the screen. However, users are advised to apply the strategies in a module for at least a couple of weeks before working on another, and the natural order of the program (by clicking “next”) leads them through a logical sequence of resources and skills.

The overall program (“My Journey”) contains five modules (“signposts”), which each includes a series of interactive tools. Tools are preceded by “guidebook” screens, which inform them about the tool and its relevance to diabetes. All tools produce a printable summary page, which can later be reviewed under the tab “What I’ve Done” at the top of the screen. In advertisement-length videos, actors illustrate key concepts such as reconceptualizing a problem. Users with low bandwidth Web connections can access the script of these videos. Throughout the program, written material is kept below a secondary school (Year 7) reading level, to maximize accessibility for users with limited education.

#### OnTrack Diabetes Signposts

**Figure 4:**
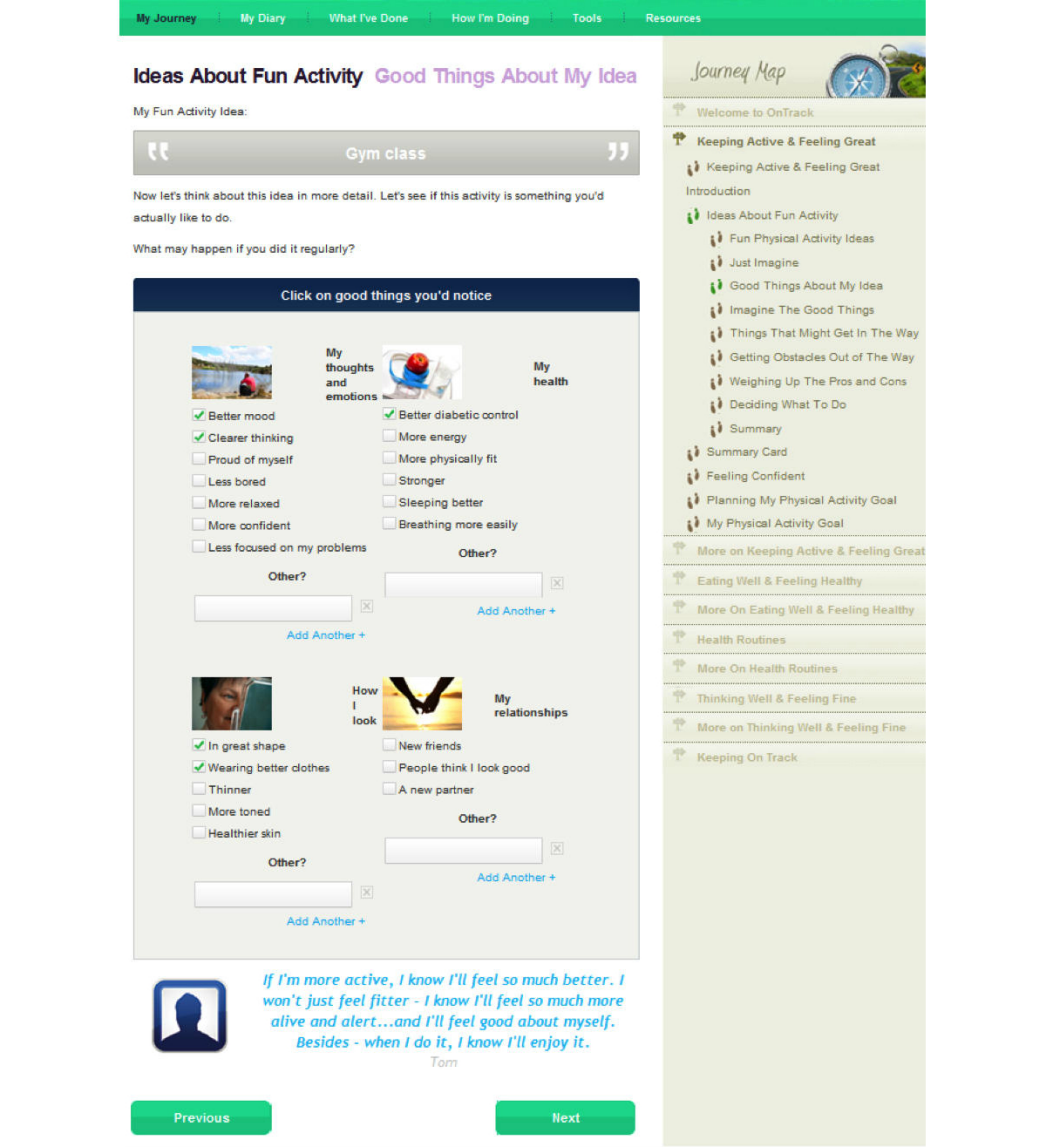
Example screen from OnTrack Diabetes: Consideration of good things about a selected physical activity.

**Figure 5:**
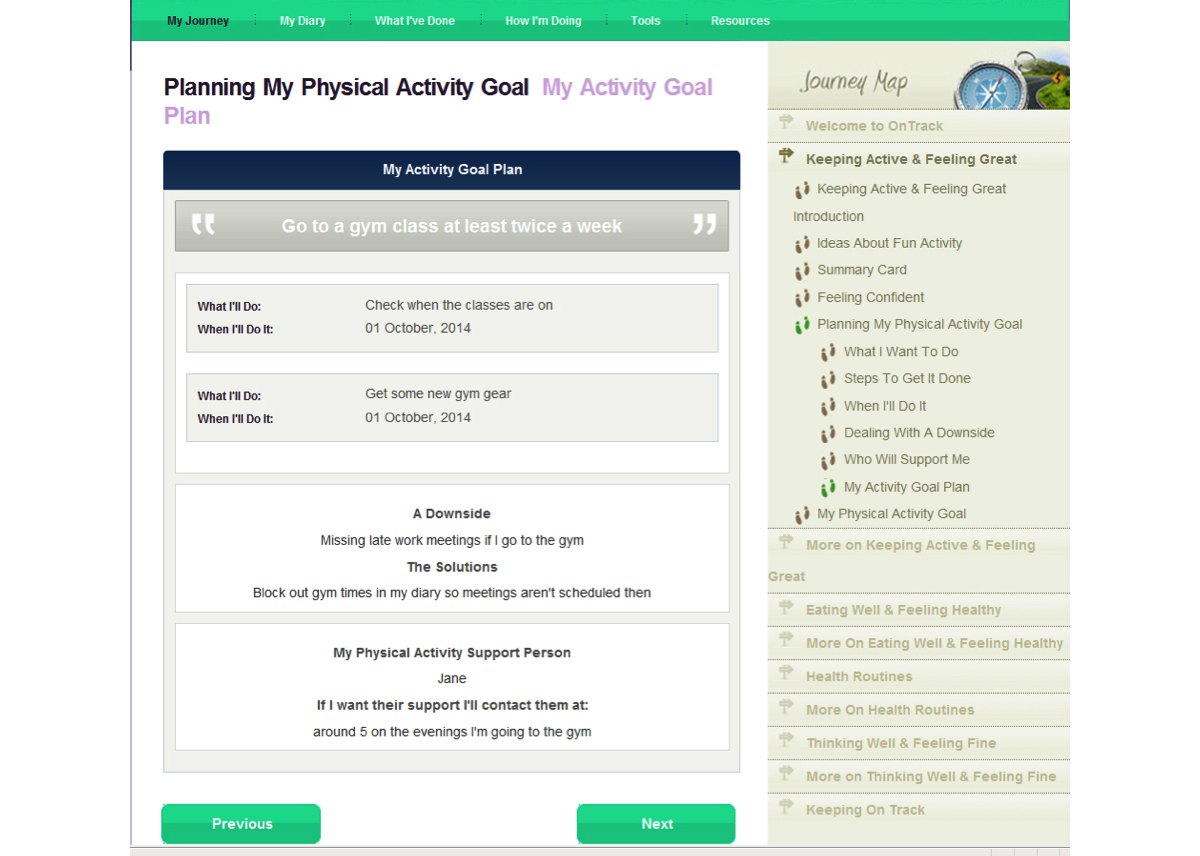
A sample summary sheet: Plan for increasing an activity.

As shown on the right side of Figure 2, the program has five signposts or modules: (1) “Keeping Active and Feeling Great”, (2) “Eating Well and Feeling Healthy”, (3) “Health Routines”, (4) “Thinking Well and Feeling Fine”, and (5) “Keeping OnTrack”. All but the last two have two sections. The first section of signposts on activity, diet, and medical regimens, asks users to select potential behavioral strategies, to imagine undertaking one, consider and image advantages of that action (Figure 4 shows this), and consider strategies to address potential barriers. Self-efficacy is boosted by a consideration of past relevant successes, and a detailed stepwise plan is formulated, including the timing of those steps and a consideration of sources of potential social support (Figure 5 shows this).

The second section of each signpost (“More on...”) contains tools that assist with making behavioral changes routine. For example, users can plan to add incidental and short bursts of activity to their week, as well as longer physical activity sessions, and specify the times and days that they will do them. It also includes a problem-solving tool [61] to assist with overcoming challenges to reaching users’ personalized goals. This tool can also be used to solve other problems, including threats to emotional well-being.

Users are asked to focus on practicing the skills learned in each section for 1-2 weeks before moving forward in the program. In the meantime they are encouraged to log on to the site regularly to self-monitor, use resources, undertake, and revisit tools as needed. The signpost “Keeping OnTrack” provides support while aiming to support the maintenance of progress. It focuses on moving on from past maladaptive behaviors and maintaining positive, new beginnings in the broader context of the individual’s life. Users are asked to evaluate positive changes since starting the program without losing sight of other life goals (eg, education, travel).

#### Self-Screening by Quizzes

There are four self-administered quizzes that enable users to evaluate their participation in diabetes self-care activities [62], mood [63], physical activity participation [64], and fat and fiber intake [65]. Self-screening enhances early recognition of distress and depression [66,67], which are important foci for ongoing assessment in diabetes patients [6], and commonly remain undetected in primary care [68]. Participants receive instantaneous, automated feedback on their results via the program, which provides a brief description of what their score indicates about how they have doing in each of the above areas.

#### OnTrack Diabetes Program Information Resources

Information resources on a number of Type 2 diabetes-related areas are included as printable fact sheets within the program. Specifically, information and resources are provided in the areas of: (1) general Type 2 diabetes information; (2) hyper- and hypo-glycaemia; (3) weight management; (4) physical activity guidelines and steps to increasing physical activity; (5) nutritional guidance including reading nutrition labels, counting carbohydrates, sugars, the glycaemic index and glycaemic load, protein, fats, fiber, dairy, salt intake, and alcohol; (6) eye care; (7) foot care; and (8) erectile dysfunction. Information sheets detail the roles of each primary care professional to diabetes management and include Web addresses to relevant organizations that allow a search for primary care professionals within any area of Australia to be performed.

#### Additional Resources, Mindfulness Resources, and Videos

The “Resources” section also contains mindfulness audios that provide spoken instructions on performing various forms of mindfulness (eg, mindfulness meditation and mindfulness of pleasure). Users are encouraged to listen to the audios on their computer or download them to an MP3 player for use offline. Guidebook pages throughout the program refer users to the most relevant mindfulness resources to each area. Inclusion of these resources is based on evidence regarding the deleterious effects of stress on glycaemic control and its tendency to increase susceptibility to dysphoria and diabetes-specific distress. Users are trained to mitigate worrying thoughts by meditative practice.

Brief videos that feature role models on key health-related and behavior change areas (eg, alcohol modification, physical activity) are also included, and provide vicarious experience.

## Results

A separate paper describes the protocol for a randomized controlled trial to provide this required evaluation [69].

## Discussion

This paper provides information on the processes involved in developing a self-guided, Web, CBT-based intervention for Type 2 diabetes self-management and dysphoria. Providing details about Web program development has implications for researchers with an interest in developing or refining current Web interventions. The focus of the current project is to provide Web-based self-guided support. Once these foundations have been evaluated, there will be scope to consider additional features that may increase its efficacy, such as the addition of a chat room or blog site to increase access to social support.

## Abbreviations

CBT: cognitive-behavior therapy
GPs: general practitioners
SCT: social cognitive theory
